# Successful cryoablation of ventricular extrasystoles originating from the vicinity of the left anterior fascicle

**DOI:** 10.1002/joa3.12304

**Published:** 2020-02-06

**Authors:** Takatsugu Kajiyama, Yusuke Kondo, Masahiro Nakano, Kazuo Miyazawa, Miyo Nakano, Tomohiko Hayashi, Ryo Ito, Haruhiro Takahira, Yoshio Kobayashi

**Affiliations:** ^1^ Department of Cardiovascular Medicine Chiba University Graduate School of Medicine Chiba Japan; ^2^ Department of Advanced Cardiorhythm Therapeutics Chiba University Graduate School of Medicine Chiba Japan

**Keywords:** catheter ablation, cryoablation, electroanatomical mapping, fascicular arrhythmia, ventricular extrasystole

## Abstract

A 32‐year‐old male received catheter ablation of frequent ventricular extrasystoles (VEs). His electrocardiogram showed monomorphic VEs with an inferior axis and early precordial transitional zone. During electrophysiological testing, a 10‐pole catheter positioned in the left ventricular outflow tract recorded sharp pre‐potentials just before the ventricular activation during VEs as well as sinus beats. Three‐dimensional mapping was performed by annotating the sharp pre‐potentials to reveal that the earliest activation site was deemed to be close to the left anterior fascicle. A cryoablation catheter was introduced into the left ventricle and freezing for 240 seconds successfully eliminated the clinical VEs without any complications.

## Graphical abstract

A 32‐year‐old male received catheter ablation of frequent ventricular extrasystoles (VEs). During the electrophysiological study, a 10‐pole catheter positioned in the left ventricular outflow tract recorded sharp pre‐potentials just before the ventricular activation during the VEs as well as sinus beats. A cryoablation catheter was introduced into the left ventricle and freezing for 240 seconds on the blue tag successfully eliminated the clinical VEs without any complications.

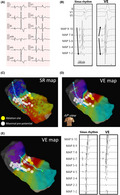

## 1 INTRODUCTION

Idiopathic ventricular arrhythmias originating from the cardiac conduction system have been well described in the literature.^1^ Catheter ablation can be an effective therapy to eliminate such arrhythmias; however, iatrogenic conduction disturbances are a major concern.^2^ In this report, we describe a successful cryoablation of frequent ventricular extrasystoles arising from the vicinity of the left anterior fascicle.

## 2 CASE REPORT

**Figure 1:**
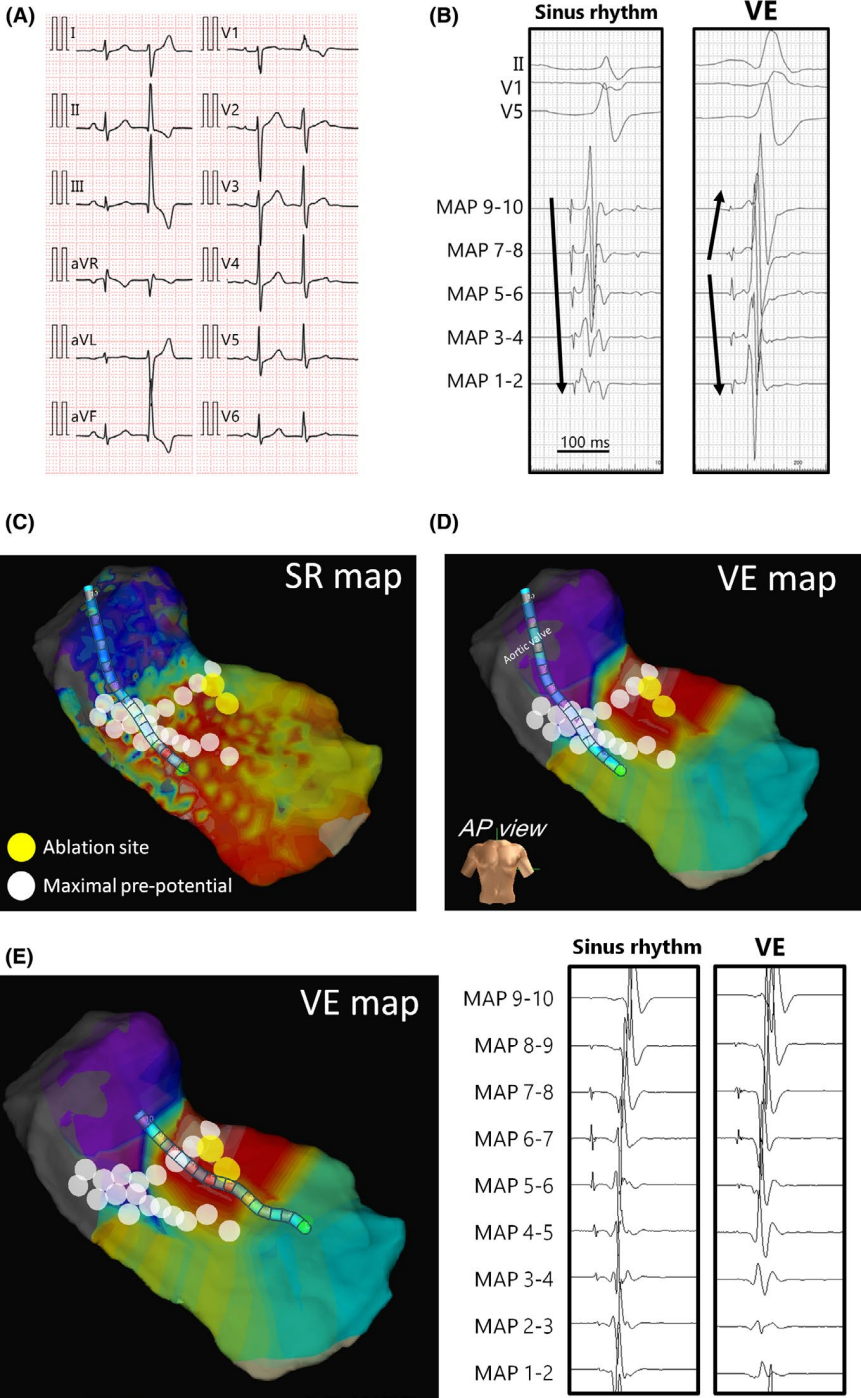
A, A twelve‐lead electrocardiography showing normal sinus rhythm with the clinical ventricular extrasystole. B, Intracardiac electrocardiography recorded via a 10‐pole catheter positioned in the subvalvular left outflow ventricular tract. Sharp fascicular potentials were observed during the clinical ventricular extrasystoles. The activation pattern of the fascicular potentials was different form that during sinus rhythm as indicated. These fascicular potentials are reflecting activation of the conduction system proximal to the left anterior fascicle. C, An activation map during sinus rhythm. D, A three‐dimensional activation map annotating the fascicular potentials during the extrasystoles. The earliest activation site (EAS) was close to the white tags, which reflected maximal amplitude of fascicular potentials during sinus rhythm. The yellow tag shows sites of cryoapplication. Of note, the position of mapping catheter was not significantly moved compared to the location during an adjacent sinus beat same as (B) and (C). E, Electrocardiograms obtained at the EAS during sinus rhythm and an adjacent ventricular extrasystole and via the 10‐pole catheter. The three‐dimensional map indicates the position of the 10‐pole catheter at the EAS

**Figure 2:**
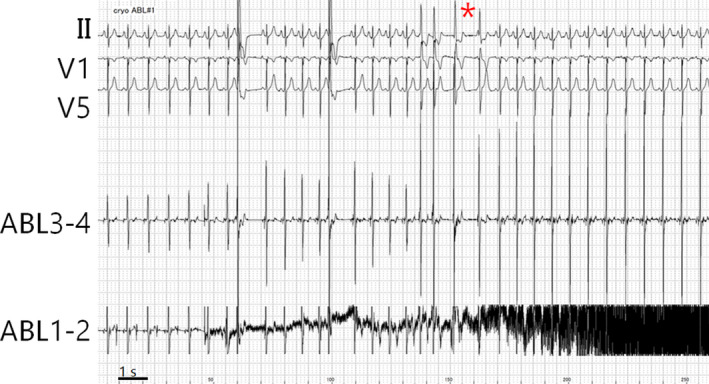
An electrocardiography just after starting the first cryoapplication. As the temperature decreased, a few clinical extrasystoles were observed (*), while the morphology of potentials recorded by the proximal couple of the ablation catheter became stable

A 32‐year‐old male presented to our clinic complaining of heart palpitations. His electrocardiography (ECG) revealed frequent monomorphic ventricular extrasystoles (VEs) interrupting normal sinus rhythm (Figure 1A). The morphology of the extrasystole exhibited a QRS duration of 105 milliseconds, inferior axis, qR pattern in the inferior leads, rS pattern in lead I, and early transition with a positive concordance in the precordial leads. Holter monitoring revealed 13, 288 VEs out of 112, 233 total heart beats. No ventricular tachycardia was recorded. The morphology suggested that its origin was in the left ventricle and was close to the native heart conduction system. Since oral bisoprolol fumarate and mexiletine hydrochloride were ineffective, catheter ablation was carried out. In the supine position, both the right femoral artery and vein were punctured after local anesthesia with a lidocaine infiltration. An 8‐French 25cm sheath was introduced into each vessel. A 5‐French pentapolar catheter (5555M‐120R, 2‐5‐2 mm spacing; Japan Lifeline) was advanced into the right ventricular apex for pacing. As for mapping, a 10‐pole 6‐French catheter (1110‐6‐2‐L1‐TE2BE2, 2‐2‐2 mm electrode spacing; IBI) was inserted into the left ventricular outflow tract (LVOT) to map the VEs in combination with a high‐density three‐dimensional mapping system (EnSite Precision; Abbott). During sinus rhythm, the 10‐pole catheter positioned in the subvalvular LVOT recorded sharp pre‐potentials reflecting the activation of the His‐Purkinje system (Figure 1B,C). During the VEs, similar pre‐potentials preceded the activation of the large ventricular potentials, while the pattern of activation differed from that during sinus rhythm (Figure 1B,D). Therefore, a local activation map was created by annotating the fascicular potentials instead of the ventricular potentials. The activation map during the VEs (VE‐map) showed that the EAS was close to an area harboring the maximal amplitude of the fascicular potentials during sinus rhythm. A fascicular potential was recorded 19 milliseconds earlier than the onset of the QRS during the VEs at the earliest activation site (EAS) (Figure 1E). To clarify the spatial distribution of the conduction system and EAS, another map referring to sinus beats (SR‐map) was created from the same data utilizing the TurboMap feature.^3^ As shown in Figure 1D, the EAS was identified in the anterior portion of the LVOT on the VE‐map. The white tags reflect the locations of the maximal His/fascicular potentials confirmed in the SR‐map. The white tags just beneath the aortic valve reflect the His‐bundle. The upper‐leftward extension after branching seemed to reflect the anterior fascicle, which was close to the EAS during the VEs. A 7‐French 4 mm‐tipped cryoablation catheter (Freezor1 S‐curve; Medtronic) was introduced into the left ventricle via the aortic valve instead of the 10‐pole mapping catheter. The ablation catheter was able to be inserted without using a U‐curve technique. After confirming a series of large potentials on the anterior fascicle, the tip was slightly moved apically, aiming to avoid any direct freezing effect on the very trunk of the conduction system. To maximize the safety, freezing in the mapping mode at −30°C was attempted; however, the temperature did not drop adequately presumably because of slippage of the catheter tip. Therefore, the ablation mode was adjusted down to −80°C and was started at the same location (yellow tag in Figure 1D). A few clinical VEs appeared just after the temperature drop (Figure 2) while no significant change in the ECG was observed throughout the freezing for 240 seconds. The fixation of the catheter tip was confirmed by the tip‐motion under cine fluoroscopy. A consolidation freeze was given for 240 seconds at that same site. There were no peri‐procedural complications including a conduction disturbance. The clinical VEs were eliminated thereafter.

## 3 DISCUSSION

In this report, we described a catheter‐based cryoablation of fascicular VEs arising from the vicinity of the left anterior fascicle. There are presumed advantages of cryoablation compared to radiofrequency (RF) ablation. First, because the catheter stability on the subvalvular left ventricular outflow tract can be suboptimal in some patients, robust adhesion to the tissue during cryoablation may lead to a more durable lesion. Significant papers have already been published, reporting that catheter‐based cryoablation is useful for VEs originating from papillary muscles containing Purkinje fibers.^4^, ^5^ Their consensus is that the stable adhesion of the cryocatheter tip on the soft structure seems to result in a good outcome. Second, conduction disturbances during cryoablation are thought to be more reversible than with RF, if the ablation is stopped immediately. This concept has already been established in the treatment of atrioventricular nodal reentrant tachycardia.^5^ In the present case, the main concern was an iatrogenic conduction disturbance such as advanced atrioventricular block, left bundle branch block, or left anterior hemiblock. Although the contact itself seemed adequate in the LVOT, a slipping effect during RF ablation may increase the chance of complications in the vicinity of the trunk of the conduction system. In contrast, during the cryoapplication in this case, the potentials recorded on the proximal electrodes of the ablation catheter remained stable without any alternation during the heart motion (Figure 2). In addition, the detailed electroanatomical mapping of the conduction system using the TurboMap also helped us to understand the precise spatial distribution of the EAS and other structures.

Disadvantages of cryoablation may also exist. Its stiff and unidirectional only catheter is more difficult to manipulate and may potentially injure the components that consist of the left ventricular outflow. The locational information of the catheter tip on the EnSite system is absent during freezing. Further studies are necessary to evaluate these advantages and disadvantages.

## 4 CONCLUSION

Cryoablation is potentially safe and effective for ventricular ectopies originating from the vicinity of the anterior fascicle.

## CONFLICTS OF INTEREST

Authors declare no conflict of interests for this article.
